# Predicting the Difficult Neonatal Airway in Fetuses With Micrognathia, Oropharyngeal or Neck Mass Lesions: Two‐Center Experience With Fetal MRI

**DOI:** 10.1002/pd.6651

**Published:** 2024-09-24

**Authors:** Stacy Goergen, James Christie, Tracy Jackson, Maria‐Elisabeth Smet, Simon Robertson, Atul Malhotra, Annie Kroushev, Mark Lovell

**Affiliations:** ^1^ Monash Imaging, Monash Health Melbourne Australia; ^2^ School of Clinical Sciences Monash University Melbourne Australia; ^3^ Department Medical Imaging The Children's Hospital at Westmead Sydney Australia; ^4^ Department of Anaesthesia Monash Health Melbourne Australia; ^5^ Westmead Institute of Maternal Fetal Medicine Westmead Hospital Sydney Australia; ^6^ Sydney Ultrasound for Women Sydney Australia; ^7^ Department of Anaesthesia Canberra Hospital Canberra Australia; ^8^ Department of Paediatrics Monash University Melbourne Australia; ^9^ Monash Newborn Monash Children's Hospital Melbourne Australia; ^10^ Monash Obstetrics Monash Health Melbourne Australia; ^11^ Department of Anaesthesia The Children's Hospital at Westmead and Westmead Hospital Sydney Australia

## Abstract

**Objective:**

Neonatal airway compromise requiring intubation, due to micrognathia or a mass lesion obstructing the fetal airway, remains difficult but important to predict prenatally. We aimed to validate MR predictors of difficult neonatal airway (DNA) in a multicentre retrospective cohort of fetuses with micrognathia and oropharyngeal/neck masses.

**Method:**

The radiology databases of two large Australian maternal–fetal medicine centers were searched for subjects meeting inclusion criteria: Pregnancies of > 18 weeks' gestation evaluated with prenatal ultrasound and MRI between 2007 and 2022 where either fetal micrognathia or a fetal cervical, oral or oropharyngeal mass was identified on prenatal ultrasound and MRI, and details of delivery/postnatal course were available including: nature of delivery, need for the fetal airway to be secured at delivery, degree of difficulty in airway securement, survival > 24 h postnatally. Imaging predictors of a difficult neonatal airway (DNA) were assessed blinded to these neonatal outcomes.

**Results:**

Twenty‐six fetuses met the inclusion criteria. Oropharyngeal and neck mass location with polyhydramnios was 100% sensitive and 82% specific for DNA. JI < 5th centile with polyhydramnios was 83% sensitive and 70% specific. JI < 5th centile with polyhydramnios was associated with DNA in 80% of cases delivered by ex utero intrapartum (EXIT) delivery and none with non‐EXIT delivery mode.

**Conclusion:**

A cervical or oropharyngeal mass with polyhydramnios predicted a difficult neonatal airway. Polyhydramnios with jaw index < 5th centile was less sensitive and less specific for a difficult neonatal airway.

**Summary**

- **What's already known about this topic?**
  ◦ Polyhydramnios suggests functional airway obstruction in fetuses with micrognathia or airway masses.
- **What does this study add?**
  ◦ Polyhydramnios was sensitive and specific for difficult‐to‐access neonatal airway (DNA) when oropharyngeal, but not lip or oral masses were present. In combination with jaw index < 5th centile, polyhydramnios was sensitive but non‐specific for DNA; routine performance of EXIT in this situation may be counterproductive because airway intubation at birth was not always needed for non‐EXIT deliveries.

## 1 Introduction

Neonatal airway compromise due to severe micrognathia or an oral cavity, oropharyngeal, or neck mass can represent an emergency at birth, particularly when undiagnosed during fetal life. It remains challenging to predict neonatal airway compromise with prenatal imaging [1]. Accessing the difficult neonatal airway (DNA) may require advanced airway techniques and specialist pediatric anesthetic, neonatal intensive care and otolaryngology expertise. The ex utero intrapartum treatment (EXIT)‐to‐airway technique is sometimes used to establish an airway before completion of delivery by maintaining placental support of the infant for up to 90 min, while securing the airway [2, 3, 4, 5, 6, 7, 8]. However, EXIT is logistically difficult, costly, and requires an expert multidisciplinary team.

Prenatal prediction of the need for, and difficulty of achieving, emergency airway support at delivery can reduce morbidity and mortality. Correct prediction relies on prenatal imaging with ultrasound (US) and magnetic resonance imaging (MRI). Semiquantitative fetal MRI‐based scoring systems, including the tracheoesophageal displacement index (TEDI), have been proposed, one for fetuses with neck masses and the other for those with micrognathia to help identify and/or quantify the “difficult” airway [9, 10]. The TEDI is the sum of the lateral and anterior displacement of the fetal trachea from the spine by a cervical mass. The studies that developed these scoring systems were performed at a single institution with small numbers of subjects, using different criteria to define “difficult,” potentially limiting generalizability. In addition, Lazar et al. [9] did not include oropharyngeal and oral masses in their assessment.

A recent single institution study of 52 fetal neck masses [11] found that polyhydramnios, tracheal deviation and compression, and anterior mass location on antenatal imaging were significantly associated with invasive airway intervention at birth, EXIT procedure, and tracheostomy. However, scoring of the difficulty in securing the neonatal airway was not addressed in this study. However, this is important in planning delivery mode and location, and thus significantly impacts the pregnant woman and her family.

The aim of our study was to perform a multi‐institutional, blinded evaluation of prenatal MRI findings to assess their diagnostic performance in predicting a DNA at delivery in a series of fetuses who had prenatally diagnosed micrognathia or an oral, oropharyngeal, tracheal/paratracheal, or cervical mass lesion.

## 2 Patients and Methods

### 2.1 Inclusion Criteria

A search of the radiology information systems and fetal MRI databases of two large combined tertiary maternity/pediatric medical centers was conducted to identify pregnancies evaluated with US and MRI in the past 15 years (2007–2022) where EITHER

1. Fetal micrognathia was diagnosed on tertiary obstetric US based on analysis of the fetal profile and measurement of an inferior facial angle less than 50°, micrognathia was confirmed on clinical grounds postnatally and the patient was referred for prenatal MRI OR
2. A fetal cervical, oral or oropharyngeal mass was diagnosed or suspected on prenatal US, confirmed with prenatal MRI and then confirmed postnatally
  AND
  Details of delivery and immediate postnatal course, including all of the following information, were available:
3. Nature of delivery (EXIT, caesarean section [LUSCS] without EXIT, normal/instrumental vaginal delivery) AND
4. Need for the fetal airway to be secured at delivery AND
5. Degree of difficulty in airway securement including technical procedures used to secure the airway AND
6. Duration of postnatal survival (≥ or < 24 h after delivery).

No patient meeting all inclusion criteria was subsequently excluded.

A waiver of full ethics application and requirement for individual patient consent was provided by our institutional ethics review committees.

### 2.2 Imaging Predictor Variables

Predictor variables were evaluated on available images by one radiologist at each institution blinded to the airway difficulty score as determined by a pediatric anesthetist (see below) as well as to the delivery mode, postnatal outcome, and any subsequent postnatal genetic diagnosis if one was reached.

Although the original MRI exam included more extensive imaging, we utilized the sagittal, axial and coronal T2‐weighted single shot echoplanar images (typically 3–4 mm slice thickness and submillimetre in plane resolution) to perform the image assessment described below. Each radiologist had access to the original radiology reports from their own institution, but in no case was data in the original clinical report used to measure/score the predictor variables.

The predictor variables of a difficult neonatal airway that were assessed were as follows:

For fetuses with masses:

1. Mass location: Oral, oropharyngeal, tracheal or cervical
2. Presence of polyhydramnios (defined as the deepest vertical pocket of amniotic fluid between the anterior and posterior uterine wall or uterine wall and placenta, not containing fetal parts or umbilical cord, measured on MRI ≥ 8 cm [12, 13, 14])
3. Maximum transverse diameter of mass
  - Tracheooesophageal displacement index (TEDI)—when measurable on the available images—for all cervical masses using the technique described by Lazar et al. [9]. This measurement was taken on an axial T2‐weighted image including the oropharynx, neck and upper mediastinum. The maximum lateral displacement of the center of the trachea from the midline (in millimeters) was added to its maximal anterior (ventral) displacement from the midline. The sum of these two measurements equaled the TEDI, that is, a total lateral plus anterior (ventral) displacement of 13 mm corresponded to a TEDI of 13.
  - Presence of indentation, deviation or compression of the oral cavity, oropharyngeal or tracheal airway (qualitative judgment, scored as yes or no).

For fetuses with micrognathia:

- Presence of polyhydramnios
- Jaw index (JI) (measurement on axial fetal MR image, as described by Tay et al. [10], with the distance in mm between the midpoint of a line joining the posterior aspects of both masseter muscles and the mandibular symphysis being divided by the bone biparietal distance to give a ratio with no units, the jaw index).
- Jaw index < 5th centile that is JI < 24 at any point in gestation (yes or no)

For all subjects

- Gestational age (GA) at MRI. If more than one prenatal MRI was performed, the GA for both MRIs was recorded and the GA at the second MRI was used for production of summary statistics.
- GA at delivery
- Biopsy diagnosis in cases of mass lesions

The two radiologists independently reviewed two cases from institution 1 (one with micrognathia and the other with lymphovascular malformation) and then compared their assessment in a videoconference meeting with screen sharing in order to reach a consensus understanding of the correct technique for assessment of the predictor variables. Due to the relatively small total number of cases, even when recruited from two institutions, these two mutually scored cases were included in the final dataset. For all other cases, independent scoring occurred. Both radiologists had more than 20 years' of prior experience in clinical fetal MR interpretation.

### 2.3 Assessed Outcome Variables

#### 2.3.1 The Key Outcome Variable Was Neonatal Airway Difficulty

##### 2.3.1.1 Indices of Airway Difficulty—Uncomplicated or Complicated

A retrospective electronic medical record review was conducted by one of three pediatric anesthetists blinded to the prenatal MR predictor scoring and prenatal imaging reports. The anesthetists who performed the review were not involved in most of the deliveries; in a single case, one of the anesthetists performing the scoring was also present at the delivery along with two other anesthetists. However, the three anesthetists performing the outcome assessments were permitted to discuss the notes with anesthetists who were present at the delivery to seek clarification of any aspect of the written records relating to airway difficulty. This was at the discretion of the scoring anesthetist, and due to the length of the study period, the anesthetist(s) present at the delivery could not always be contacted for assistance with clarification. A single case was reviewed by a single anesthetist, that is interobserver agreement on airway scoring was not assessed.

A complicated and uncomplicated airway at birth was defined as previously by Lazar et al. [9]:

An *uncomplicated airway at birth:*

- unobstructed spontaneous respirations or
- relatively easy intubation using direct laryngoscopy alone

A *complicated airway at birth*:

- difficult intubation using direct laryngoscopy (the need for significant manual pressure on the neck or mass to bring the vocal cords into view) OR
- multiple attempts at intubation OR
- the need for
  - airway instrumentation with rigid bronchoscopy OR
  - tracheostomy
- respiratory‐failure related death < 24 h of delivery following unfeasibility of airway access or decision for palliative/comfort care at delivery with no attempt at intubation.

**We also collected data on fetal survival post delivery (< 24 h or ≥ 24 h) and delivery mode, the latter being classified into one of four categories:**

- EXIT
- Caesarean (LUSCS), intubation required within 1 h of delivery
- LUSCS, no intubation required within 1 h of delivery
- Vaginal delivery

### 2.4 Data Analysis

Radiologist and anesthetist raters recorded data in a password‐protected Excel spreadsheet (Microsoft Corporation, Redmond, Washington, U.S.A.). Summary statistics for each predictor variable were generated and, where feasible, binary predictors of the key outcome of “difficult airway” (polyhydramnios, JI < 5th centile, TEDI > 12, airway deviation) were compared with chi‐square or Fisher's exact test. Continuous variables (maximum mass diameter, TEDI, JI) were compared with the Wilcoxon rank sum or unpaired *t*‐test as appropriate. Multivariable logistic regression was not feasible due to the number of subjects and predictor variables.

## 3 Results

Twenty six fetuses met the inclusion criteria. All but one were singletons; one of a pair of diamniotic dichorionic twins had mandibular epignathus. Details of individual cases are provided in Supporting Information S1.

### 3.1 Mass Lesions

Ten of the 26 infants had masses. These included: Three cervical lymphovascular malformations, one cervicofacial teratoma; one multifocal rhabdoid tumor with oropharyngeal and tracheal compression; two cases of epignathus; one oral duplication cyst; one ranula; and one case of Beckwith–Wiedemann syndrome with severe macroglossia.

Two infants died in the neonatal period: one with multiorgan failure complicating disseminated rhabdoid tumor that did not respond after 2 weeks of chemotherapy and the other with a large craniocervical/facial teratoma leading to airway obstruction‐related death within the delivery suite. A third infant with a large lymphovascular malformation died at 2 months of age due to infective complications of sclerotherapy and surgery for the lesion.

**FIGURE 1:**
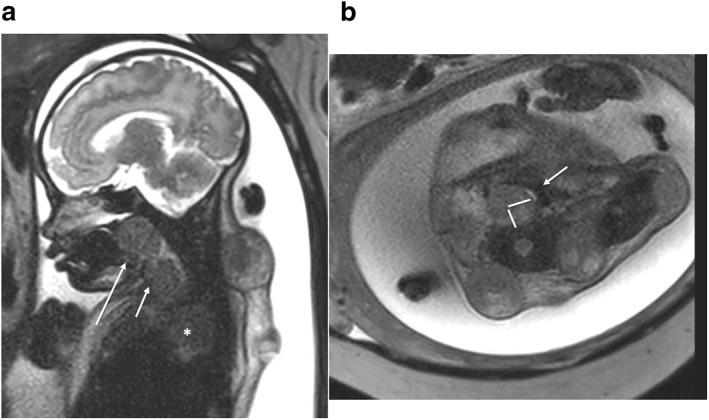
Fetus at 37 weeks of gestation with a disseminated rhabdoid tumor. (a) Sagittal T2‐weighted single‐shot EPI image demonstrates polyhydramnios and posterior oral cavity/oropharyngeal, (long arrow) paratracheal, (short arrow) and posterior mediastinal (*) as well as posterior nuchal mass lesions. (b) Axial T2‐weighted single‐shot EPI image demonstrating measurement of the tracheoesophageal displacement index (indicated by perpendicular lines drawn on the image) comprised of the sum of the transverse and anterior displacements, in millimeters, of the compressed, slit‐like trachea (arrow) away from the spine (in this case the TEDI was 20).

Five EXIT procedures were performed. All five pregnancies had polyhydramnios and 2 of 5 were not difficult intubations; one had a cervical lymphovascular malformation and the other had a large epignathus originating from the mandible but attached by a narrow pedicle, allowing it to be easily displaced by the anesthetist to permit intubation. The pedicle and mobility were not identifiable on the fetal MR images (Figure 1a,b).

The other five infants who did not have EXIT did not require intubation; 4 of the 5 (cases of Beckwith–Wiedemann syndrome, epignathus, duplication cyst, and ranula) breathed spontaneously. The fifth non‐EXIT delivery was the neonate with large craniocervical/facial teratoma with intracranial extension demonstrated by prenatal US and MR; following multidisciplinary prenatal prognostic counseling, the infant's parents had requested palliation and “comfort care” after delivery with no attempts made to access/secure the airway.

**FIGURE 2:**
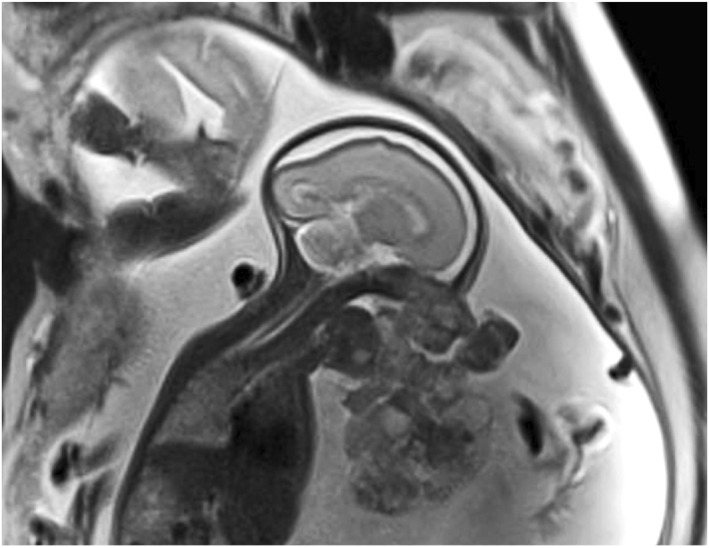
One fetus of a dichorionic diamniotic twin pair at 26 weeks' of gestation with mandibular epignathus. Sagittal T2‐weighted single‐shot EPI image demonstrates polyhydramnios and a large, lobulated mandibular/anterior oral cavity mass diagnosed prenatally as epignathus. At EXIT delivery, the mass was found to be attached to the mandible by a pedicle and could easily be displaced by the anesthetist to enable intubation.

Table 1 provides predictor variables and sensitivity and specificity for DNA. The best predictor of a difficult airway was a “caudal” (oropharyngeal, tracheal or cervical) mass lesion (Figure 2). In all four cases of a caudal lesion location, however, polyhydramnios was also present.

**TABLE 1:** Imaging predictors of airway difficulty in fetuses with oral, oropharyngeal and cervical mass lesions.

*N* = 10	Airway difficulty + (*N* = 4)	Airway difficulty − (*N* = 6)	
Polyhydamnios (present in *n* = 6)	4 (67%)	2 (33%)	Sens = 100% Spec = 66%
TEDI (assessable in *n* = 8)	8 mm (4–11)	7 mm (2–9)	*p* = 0.36
Location			
Oropharynx/tracheal/cervical +/− lip or oral involvement	4	1	Sens = 100% Spec = 81%
Lip/oral only	0	5	
Airway compression (*n* = 8)	4	4	Sens = 100% Spec = 50%
Airway compression + polyhydramnios (*n* = 6)	4	2	Sens = 100% Spec = 66%
Oropharynx/trachea/cervical + polyhydramnios	4	1	Sens = 100% Spec = 81%
Median diameter of measurable^a^ mass in millimeters (range) (*n* = 9)	81 (range 70–120)	51 (range 16–86)	*p* = 0.18
Gestational age at MR^b^ (weeks)	34 (range 29–34)	32 (range 27–36)	*p* = 0.28
EXIT (*n* = 5)			

Abbreviations: JI = Jaw index; Poly = polyhydramnios (deepest vertical pocket > 8 cm); Sens = sensitivity; Spec = specificity; TEDI = tracheoesophageal displacement index.

^a^ If more than one MR was performed, the gestational age at the last study was used.

^b^ In the case of Beckwith–Wiedemann syndrome, obstruction was due to severe macroglossia, so the mean diameter of a “mass” was not measurable.

### 3.2 Micrognathia

**FIGURE 3:**
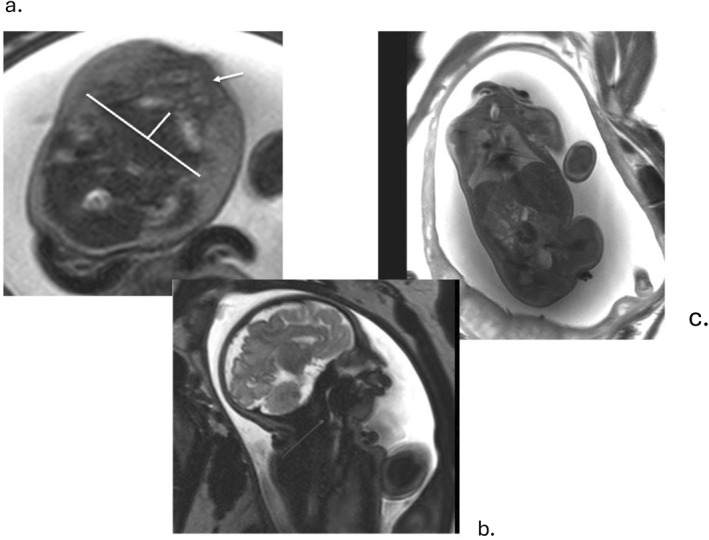
Fetus at 36 weeks' gestation with micrognathia and cerebrocostomandibular syndrome due to an autosomal dominant de novo *SNRPB* pathogenic variant. (a) Axial T2‐weighted single‐shot EPI image demonstrating polyhydramnios and measurement of the mandibular depth, the numerator of the jaw index. Arrow indicates the alveolar ridge of the palate; note the posterior position of the mandible relative to the palate. (b) Sagittal T2‐weighted single‐shot EPI image demonstrating glossoptosis, resulting in marked narrowing of the oropharyngeal airway due to absence of the posterior palate consistent with Pierre Robin sequence. (c) Coronal T2‐weighted single‐shot EPI image showing bell‐shaped fetal chest due to rib deformities.

**FIGURE 4:**
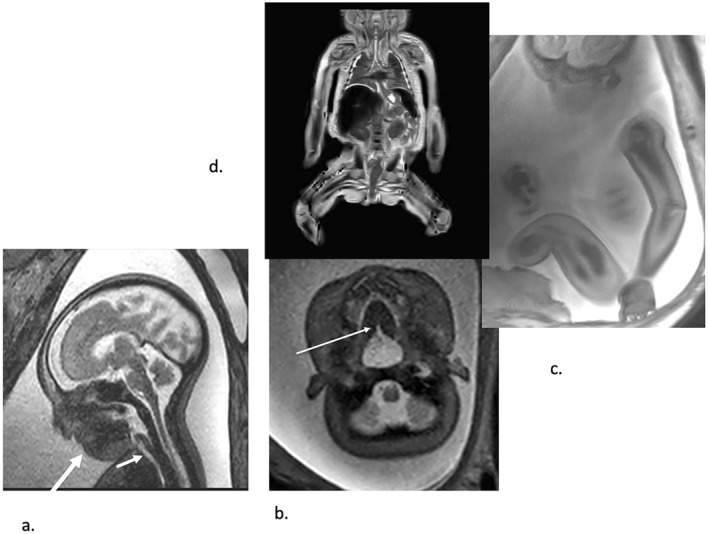
Fetus at 36 weeks' gestation with biopsy‐proven Type 2 muscle fiber deficiency. (a) Sagittal T2‐weighted echoplanar image showing abnormal fetal profile due to micrognathia (long arrow) and there is polyhydramnios but the jaw index was greater than the 5th percentile at 33. There is also no evidence of glossoptosis or oropharyngeal or tracheal (short arrow) obstruction. (b) A very small posterior palate cleft is seen on this axial T2‐weighted echoplanar image (arrow). (c) Sagittal T2‐weighted echoplanar image demonstrating gross muscular underdevelopment in the arm and thigh, confirmed on coronal T2‐weighted fast spin echo post‐mortem MRI image (d). Polyhydramnios was due in this case to neuromuscular dysfunction rather than micrognathia‐related airway obstruction.

Sixteen infants had micrognathia, 13 of 16 having the Pierre–Robin sequence (PRS) confirmed postnatally. Of the 16, 3 had chromosomal microarray abnormalities and 5 were diagnosed with skeletal dysplasia with two cases of cerebrocostomandibular syndrome (Figure 3a–c), one Stickler syndrome, one Nager syndrome and one case of as yet genetically undiagnosed skeletal dysplasia. There was one case of type 2 smooth muscle fiber deficiency, but no explanatory genetic abnormality was found on postnatal whole‐exome sequencing (Figure 4a–d).

There were 6 EXIT procedures; all had JI < 5th centile and were diagnosed with the Pierre–Robin sequence either pre‐ or postnatally. In 5 / 6 cases delivered by EXIT, the JI was < 5th centile and polyhydramnios was present; in 4 of these 5, a DNA was encountered. The fifth case did not have a DNA but had Stickler syndrome. The other EXIT procedure, involving a fetus without polyhydramnios ultimately diagnosed with skeletal dysplasia, had uncomplicated intubation at the time of delivery.

Of the 10 cases of micrognathia *not* delivered by EXIT, 3/10 had the combination of JI < 5th centile and polyhydramnios; 2 of these 3 did not need intubation at birth but did require intubation within 2 h of delivery despite decubitus nursing and less invasive measures such as CPAP. These two infants had

– Nager syndrome with difficult intubation
– Congenital diaphragmatic hernia and easy intubation

The third infant had PRS but no syndromal features identified postnatally, and normal chromosomal microarray and whole‐exome sequencing not performed. This infant also did not require intubation at birth but did require support with a nasopharyngeal airway shortly after birth and decubitus nursing while in the neonatal intensive care unit until day 12 of life when these were no longer needed to support oxygenation.

The other seven patients not delivered by EXIT did not require immediate postnatal intubation, but one had difficult intubation for all perinatal surgical procedures and had diastrophic dysplasia, a JI < 5th centile but no polyhydramnios.

Table 2 lists the predictor variables and outcome for fetuses with micrognathia. The best predictor of a difficult airway was a jaw index < 5th centile combined with polyhydramnios, which had sensitivity and specificity of 83% and 70%, respectively. In six fetuses with JI > 5th centile, only one had neonatal airway difficulty, whereas 50% of 10 fetuses with JI < 5th centile did. Polyhydramnios was 100% sensitive for DNA but had a specificity of only 60%.

**TABLE 2:** Imaging predictors of airway difficulty in fetuses with micrognathia.

*N* = 16 (PRS in *N* = 13)	Airway difficulty at birth + (*N* = 6)	Airway difficulty at birth − (*N* = 10)	
Poly (*n* = 10)	6	4	Sens = 100% Spec = 60%
JI median (range)	18 (16–27)	24.5 (18–27)	
JI < 5th centile (*n* = 10)	5	5	Sens = 83% Spec = 50%
JI < 5th centile + poly (*n* = 8)	5	3	Sens = 83% Spec = 70%
JI < 5th centile OR poly (*n* = 12)	6	6	Sens = 100% Spec = 40%
Gestational age (weeks) at delivery^a^	36.5 (36–41)	37.5 (36–40)	

Abbreviations: JI = Jaw index; Poly = polyhydramnios (deepest vertical pocket > 8 cm); Sens = sensitivity; Spec = specificity.

^a^ If more than one MR was performed, the gestational age at the last study was used.

## 4 Discussion

Our study has confirmed the sensitivity of polyhydramnios for identification of the fetus at high risk of DNA at delivery when severe micrognathia or an oropharyngeal or cervical mass is present. However, polyhydramnios has low specificity as a *single* predictor of a difficult airway in the fetus with a lip or oral cavity mass or JI < 5th centile.

We have identified caudal (to the lips and oral cavity) mass lesions in conjunction with polyhydramnios as a sensitive *and specific* predictor of a DNA. Death in the neonatal period or early infancy occurred in nearly 1 in 3 infants in this situation. However, our study cohort was limited by the fact that fetuses with “caudal” mass lesions all had polyhydramnios. Therefore, we are unable to determine whether a “caudal” mass even without polyhydramnios is predictive of DNA because we had no such cases.

We also found that subjective assessment of airway obstruction or narrowing by a mass anywhere from the lips to the trachea in combination with polyhydramnios was no more sensitive, and was also less specific, for DNA than was mass position in combination with polyhydramnios. Although the TEDI was not significantly different between fetuses with and without a DNA, this may reflect both small sample size and also a non‐neck location of many of the masses in our cohort, precluding use of the TEDI.

In our fetuses with micrognathia, polyhydramnios alone was 100% sensitive for a DNA, unlike Tay et al. who found this sign to be only 63% sensitive. However, it was poorly specific (60%) in our series. Like Tay et al. we found the combination of JI < 5th centile OR polyhydramnios to have 100% sensitivity but poor specificity (in our case, 40% and for Tay et al. 50%).

The combination of JI < 5th centile AND polyhydramnios was less sensitive (83%) but more specific (70%) for a DNA. Of the eight infants with this combination of abnormalities, five were delivered by EXIT and in 4/5 (80%) there was a DNA; however, the other three, all non‐EXIT deliveries, did not need intubation, and thus did not have a DNA at the time of delivery. When fetal JI was > 5th centile, only 1 of 6 infants had a DNA but this infant also had polyhydramnios, related to a severe neuromuscular disorder with poor sucking and swallowing. The two infants with JI < 5th centile but no polyhydramnios did not have a DNA.

These findings raise clinically relevant issues related to delivery planning for the fetus with micrognathia:

- *Absence* of polyhydramnios was sensitive in our cohort for predicting *lack* of DNA regardless of JI.
- A slight majority of infants with JI < 5th centile AND polyhydramnios will have DNA, but EXIT might paradoxically increase this risk by “forcing” intubation because the baby is born anaesthetized and the supine position maximizes posterior tongue displacement, making the airway harder to visualize and secure. When intubation is not *required* at delivery, because EXIT has not been performed, the combination of JI < 5th centile and polyhydramnios may not be predictive of DNA based on our results. This observation likely reflects the position‐dependent nature of the airway obstruction in micrognathia, enabling it to be managed successfully in many cases with decubitus positioning and minimal airway support (e.g., placement of a nasopharyngeal airway or positive pressure ventilation). However, events during the labor and delivery that might necessitate resuscitation, and thus intubation, are unpredictable by fetal MRI. Therefore, whether this risk is better managed with EXIT delivery where there is extra time to secure the airway, despite DNA being likely, or specialized multidisciplinary airway expertise and equipment on hand at non‐EXIT delivery should be determined by local factors, available expertise and multidisciplinary planning.
- Due to our study design, we cannot determine whether EXIT or alternatively non‐EXIT delivery with expert airway support and equipment in the delivery room is the “better” method of delivery in terms of morbidity, mortality and cost in the fetus with severe micrognathia with or without polyhydramnios. Furthermore, obstetric considerations will factor into decision‐making. One of our three non‐EXIT deliveries of a fetus with PRS, jaw index less than the 5th centile and polyhydramnios was a primigravida and the patient and her husband decided on labor induction and a vaginal delivery with airway management expertise immediately available following extensive multidisciplinary counseling about the risks and benefits of this approach.
- When the JI is > 5th centile, but there is polyhydramnios, causes other than micrognathia, such as neuromuscular disorders, should be considered.

Multicenter prospective studies are needed to inform clinical practice about the optimal delivery method for the fetus with JI < 5th centile and polyhydramnios. Although randomized controlled trial evidence would be optimal and is likely ethical in view of the equipoise on this issue, the relative rarity of the clinical situation makes this unlikely. Therefore, prospective collection of clinical data with standardized neonatal outcome metrics, imaging predictors and definition of airway difficulty, is needed.

Fetal MR should be considered in every fetus with polyhydramnios and sonographic evidence of an anterior palate cleft or micrognathia because of the frequent difficulty in confirming PRS with US alone [15]. MRI helps assess the severity, cause, and anatomical details of airway compromise [9, 16]. Its advantages over US include lack of shadowing of the oral cavity, oropharynx and trachea by the mandible and alveolar plate and more accurate diagnosis of Pierre Robin sequence (PRS) because of better visualization of the posterior palate. The larger field of view and the superior contrast resolution afforded by MRI facilitate delineation of the nature and full extent of mass lesions, including intracranial involvement by facial teratoma [9, 10, 17, 18]. In our experience, MR following US provided helpful anatomical information at the multidisciplinary delivery planning meetings we held.

Our study has important limitations:

1. Retrospective review of documentation about “how difficult” the airway is limited by variable detail in clinical notes. We also did not measure agreement between two anesthetists about airway difficulty scoring.
2. Dichotomization of the airway as “easy” or “difficult,” despite clear criteria for each being used, risks loss of granularity, for example exactly how many minutes it took for the airway to be secured and which instrument or procedure (e.g., tracheostomy, rigid bronchoscopy) was required to achieve this.
3. We were unable to control for the chief confounder of DNA: variation in anesthetist skill. However, this may make our results more generalizable.
4. Retrospective design meant we were unable to determine why EXIT delivery was chosen in some cases and not others.

**FIGURE 5:**
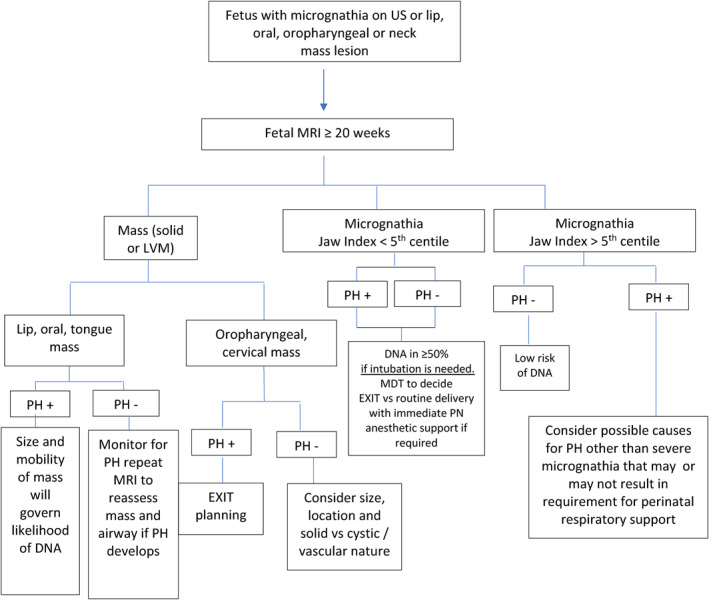
Fetal MR—Based decision algorithm for delivery planning for the fetus with micrognathia or an oral, pharyngeal, or neck mass. DNA = difficult neonatal airway; EXIT = ex utero intrapartum‐to‐airway therapy; JI = jaw index; LVM = lymphovascular malformation; PH = polyhydramnios; US = ultrasound.

However, at our institutions, the management of delivery of a fetus with potential for a difficult neonatal airway is a stepwise multidisciplinary decision‐making process beginning with prenatal US to identify the potentially at risk fetus. This process is summarized in Figure 5.

In essence, “caudal” airway masses (i.e., oropharyngeal and cervical location) in combination with polyhydramnios are routinely managed with EXIT at our institutions because of the expectation of “fixed,” that is, position‐independent airway obstruction. We did not have a case of a lip, tongue, or oral cavity lesion associated with DNA nor did any fetus without polyhydramnios but with micrognathia of any severity have a DNA so we regard these situations as low risk based on our data. Optimal delivery management of the combination of JI < 5th centile with polyhydramnios (i.e., EXIT vs. no EXIT but with airway expertise and instruments in the delivery room) is best determined by multidisciplinary consideration of local capability, logistical considerations and the wishes of the pregnant patient.

5 Some types of neck masses, such as thyroid goiter, were not represented amongst our cases. The single case in our series of an oropharyngeal/cervical mass lesion associated with polyhydramnios where airway access (at EXIT) was not difficult was a lymphovascular malformation. Lazar et al. [9] found that teratoma histology was independently associated with airway difficulty but not reflected in a higher TEDI due, presumably, to rigid fixation rather than displacement of the trachea by the mass. However, this association of teratoma histology with DNA is likely location‐dependent as our case of facial teratoma was not associated with DNA.

In conclusion, this study adds to existing knowledge by confirming the sensitivity and specificity of polyhydramnios in fetuses with oropharyngeal and neck masses as a predictor of DNA but highlights limitations of its use in the prenatal prediction of DNA in fetuses with micrognathia in whom airway obstruction is more dynamic and position‐related. Our work may help inform shared decision‐making with pregnant women about the risks and potential benefits of different options for delivery, including EXIT.

## Ethics Statement

Our institutional ethics committees provided a waiver of the requirement for individual patient consent and full ethical review based on the retrospective study design and lack of patient inconvenience or harm.

## Consent

Individual patient consent was not obtained for this study.

## Conflicts of Interest

The authors declare no conflicts of interest.

## Supporting information

Supporting Information S1

## Data Availability

More detailed patient level data is available from the corresponding author upon request.
