# Supplementary material for: Predicting the Difficult Neonatal Airway in Fetuses With Micrognathia, Oropharyngeal or Neck Mass Lesions: Two‐Center Experience With Fetal MRI
Source: Prenat Diagn. 2024 Sep 24;44(13):1593–602. doi: 10.1002/pd.6651 (PMC11628208; doi:10.1002/pd.6651)
Supplement: Supplementary file 1 — Supporting Information S1 [file PD-44-1593-s001.pdf]

Supplementary Table: Clinical and Imaging Data for Individual Cases: Masses and Micrognathia

			Diagnosis	estational age at MR (weeks)	Gestational age at delivery	MR to delivery time elapsed (weeks)	Airway Difficulty (Yes = 1, No = 0)	Mode of delivery (EXIT = ;, LUSCS need for intubation within 1 hour of delivery = 2; LUSCS no need for intubation = 3; Vaginal delivery no need for immediate intubation = 4)	Survival > 24 hours (Y = 1, N = 0)		polyhdramnios defined as DVP > 8mm (Y = 1 N = 0)	Deepest vertical pocket of amniotic fluid (cm)	Mass = 1 Micrognathia = 2	Jaw Index	JI<5th centile (JI <24) (Y = 1 N = 0)	if mass longest axial diameter mm	measure TEDI*	is there subjective evidence of airway indentation (from mouth to hypopharynx), tracheal shift or tracheal compression? Y = 1 N = 0	location of mass : oral cavity = 1, pharynx = 2, paratracheal = 3, lip / mouth = 4	Notes on imaging	
1			Epignathus / facial teratoma	27	DCDA Twins 30.5 weeks	3.5	**0**	**1**	**1**	0 - no notes on intubation, Apgars 2, 6, 7. Anaesthesist not mentioned as present but Richard Barnes there for C/S. Nasal tube therefore likely easy	1	14	1	29	0	86	2	1	1	oral cavity mass producing glossoptosis	
2			Epignathus	29	41	12	0	4	1	0 -No comment but NVD, Apgars6, 9. No notes from baby from 2009	0	6	1	34	0	16	not measurable because absent chest imaging	0	4		
3			LVM	33	37	4	0	1	1	0 - First pass. Not obvious that which blade used but stated uncomplicated.	1	10	1	29	0	80	9	1	2,3	very large mass but no retrotracheal extension and bilateral so TEDI not much increased. Hypopharyngeal indentation	
4			LVM	34	37	3	1	1	1	1- direct laryngoscopy challenging using bougie	1	9	1	35	0	120	not measurable because suboptimal plane not perpendicular to axial plane of trachea	1	2,3	indentation posterior pharynx and hypopharynx	
8			rhabdoid tumour	37	37	0	1	1	1	1 - needed Lopro glidascope and bougie	1	13	2	38	0	43 (paratracheal mass) 28mm (oropharyngeal mass)	11	1	2, 3	oropharyngeal and paratracheal compression	
16			Ranula	32	40	8	0	4	1	No intervention required at birth, Cystic structure floor of the mouth occupying 75% of oral cavity. Cyst 25X26X36mm on MRI. Subsequent intubations easy.	0	7.5	1	29	0	35	8	1	1	oral cavity narrowed, simple unilocular cyst final dx Ranula on histo	
17			Beckwith Wiedemann	36	37	1	0	3	1	Tongue protruding from mouth at birth, but no intervention required after delivery. Possible Beckwith - Wiedeman Syndrome. Subsequent intubatiomns uneventful	0	50	2	26	0		8	0	1	big tongue prutrudes out of mouth Beckwith Weidemann	
24			Cervical teratoma	29	31	2	Unknown	4	0	Delivered vaginally, no attempt made to intubate. The child had large right anterior neck mass. Baby palliated with morphine. Died 12 hours after birth. No assessment obtained as to the difficulty of intubation.	1	110	1	not measuarable		100	4	1	1,2,3	palliative care at delivery	
26			LVM	34	38	4	1	1	1	Lengthy laryngoscopy - almost 90 seconds looking at the video. Bougie into trachea and railroaded ETT. Chest rise confirmed with BVM prior to cord crossclamp. Times: Uterine incision 1443, Head delivered 1448, ETT placed 1450, Cord clamped 1451	1	11	1	23	1	70	11	1	2,3	LVM	
18			Duplication cyst	32	39	7	0	3	1	Sublingual cyst, 32X23X12 mm. Tongue displaced anteriorly, no intervention required at delivery. Subsequent intubations not difficult.	0		1	36	0	35	9	1	1	Unilocular cyst Histo = duplication cyst (JAMES: A DUPLICATION CYST IN ORAL CAVITY OR IS CODE FOR LO	
5			Micrognathia PRS 1p36.1	32	38	6	0	3	1	0 - apgars 9, 10. Spont vent, some mask CPAP	0	6	2	27	0	na	3	1	na	oral cavity obstruction, no tracheal obstruction	
6			Micrognathia, PRS due to cerebrocostomandibular syndrome	34	36	3	1	1	1	1 - multiple attempts, Lopro	1	10	2	18	1	na	2	1	na	oropharyngeal and oral cavity indentation	
7			Micrognathia and type 2 smooth muscle deficiency	24	36	12	1	4	1	1 - failed intubation in delivery suite, mulitple attempts at intubation during nicu resuscitation eventually succeeded. Anaesthetics not involved.	1	16	2	27	0	na	2	0	na		
9			Micrognathia and PRS	35	40	5	0	4	1	0- laryngeal mask used and baby turned on side. Immediate desaturation when on back but intubation not required so degree of difficulty not assessable	1			18	1	na	2	1	na	baby nursed on side and nasopharyngeal airway used but no intubation needed	
10			Cerebrocostomandibular syndrome	29 and 33	36	3	1	1	1	Three attempts at intubation during EXIT, eventual tracheostomy on mother during EXIT by ENT. Cerebrocostomandibular syndrome. Mechanical ventilation for three weeks post delivery. Child now 6yo.	1	13.7	2	16	1		15	1		marked upper airway narrowing, big posterior cleft	
11			PRS	32 and 36	37	1	0	1	1	Uneventful intubation with EXIT using videolaryngoscope, subsequently micrognathia,cleft palate,mandibular distractions.	1	13.2	2	23	1		trachea not clearly identified on acquired axial images due to motion	1		oral compression cleft palate final dx Stickler syndrome	
12			Micrognathia	30	40	10	0	3	1	Vigorous at birth, not requiring intubation, CPAP initially, subsequent intubations not difficult.	0	6.8	2	24	0		4	1		Cleft + micrognathia final dx Pierre Robin	
13			CDH micrognathia	30	37	7	0	4 R:RVaginal delivery, needed to be intubated immediately, however easy intubation.	1	Diaphragmatic hernia and mild micrognathia, subsequently easy intubation.	1	14.7	2	23	1		5	1		CDH mild micrognathia possible cleft on MRI	
14			PRS also deletion 7p14.3 to 7p14.1	34	38	4	0	4 Vaginal delivery, no need for intubation initially.	1	Hypotonia, miocrognathia, subsequent intubations not difficult.	1	8.3	2	27	0		6	1		moderate narrowing bilateral cleft PR sequence	
15			micromelia micrognathia, bilateral cleft (PRS?)	36	37	1	0	1	1	Micromelia, micrognathia, skeletal dysplasia, Subsequent intubations not difficult.	0	7.5	2	22	1		7	1		moderate to marked oral cavity narrowing Bilateral cleft	
19			Diastrophic dysplasia and PRS	32	37	5	0	2	1	Diastrophic dysplasia, Put on side with nasopharyngeal airway and managed with CPAP. Not inubated at birth but expectation was that intubation would likely be difficult. Susequent intubations for LBO's extremely difficult (Grade iV) requiring ENT assistance. Somewhat easier intubation with videolaryngoscope but still very difficult.	0	6	2	21	1		8	1		tongue elevated, cleft palate PRS	
20			Nager syndrome and PRS	31	41	10	1	2	1	Initial put on CPAP but requiring intubation 47 minutes after deivery. Difficult airway - intubated after 4th attempt. Grade IV airway , then remained difficult to intubate with subsequent intubations despite mandibular distractions. Nager syndrome.	1	146	2	16	1		7	1		big central cleft tongue up	
21			PRS	33	40	7	0	4	1	not requiring intubation at birth.	0	55	2	25	0		5	1		tongue elevated, cleft palate PR sequence	
22			PRS	31	36	5	0	4 (but needed intubation 15 mins c	1	Initial mask ventilation, but needed intubation after 15 mins. Not noted as difficult intbation. #2.5 ETT required, unable to place #3.0. Subseuqent intubations not noted as difficult.	0	35	2	26	0		6	1		cleft, tongue up PR sequence	
23			16p 11.2 deletion and PRS	34	38	4	1	1	1	Intubated after 6 mins via videolaryngoscope during EXIT procedure. Baby required atropine and adrenaline for foetal bradycardia. Subsequent intubations not difficult for other anaesthetics.	1	105	2	18	1		5	1		cleft + ? Choanal atresia PR sequence	
25			PRS and multiple hemivertebrae, no syndromal diagnosis yet	29	37	8	1	1	1	EXIT. Two anaesthetists used for intubation. Initially used CMAC #0 Macintosh blade, view difficult, tongue in the way. Then used #0 Miller blade view of base of cords with difficulty. Cricoid utilsed. Then 2.8 flexible bronchoscope through right nostril and larynx located and bronchoscope through	1	13	2	21	1		6	1			
Legend	lymphovascular malformation																				
	lower uterine caesarean section																				
	not applicable																				
	Pierre Robin sequence																				
